# Parental and professional perceptions of informed consent and participation in a time-critical neonatal trial: a mixed-methods study in India, Sri Lanka and Bangladesh

**DOI:** 10.1136/bmjgh-2021-005757

**Published:** 2021-05-21

**Authors:** Stuti Pant, Maya Annie Elias, Kerry Woolfall, Maria Moreno Morales, Bensitta Lincy, Ismat Jahan, Samanmali P Sumanasena, Siddarth Ramji, Seetha Shankaran, Sudhin Thayyil, HELIX Trial consortium investigators, Arasar Seeralar

**Affiliations:** 1 Centre for Perinatal Neuroscience, Imperial College London, London, UK; 2 Perinatal Trials Unit Foundation, Bengaluru, India; 3 Institute of Population Health, University of Liverpool, Liverpool, Merseyside, UK; 4 Perinatal Trials Unit Foundation, Chennai, India; 5 Department of Neonatology, Bangabandhu Sheikh Mujib Medical University, Dhaka, Dhaka District, Bangladesh; 6 Disability Studies Department, University of Kelaniya, Kelaniya, Sri Lanka; 7 Pediatrics, Maulana Azad Medical College, New Delhi, Delhi, India; 8 Neonatal- Perinatal Medicine, Wayne State University, Detroit, Michigan, USA

**Keywords:** child health, public health, qualitative study, randomised control trial, clinical trial

## Abstract

**Introduction:**

Time-critical neonatal trials in low-and-middle-income countries (LMICs) raise several ethical issues. Using a qualitative-dominant mixed-methods design, we explored informed consent process in Hypothermia for encephalopathy in low and middle-income countries (HELIX) trial conducted in India, Sri Lanka and Bangladesh.

**Methods:**

Term infants with neonatal encephalopathy, aged less than 6 hours, were randomly allocated to cooling therapy or usual care, following informed parental consent. The consenting process was audio-video (A-V) recorded in all cases. We analysed A-V records of the consent process using a 5-point Likert scale on three parameters—empathy, information and autonomy. In addition, we used exploratory observation method to capture relevant aspects of consent process and discussions between parents and professionals. Finally, we conducted in-depth interviews with a subgroup of 20 parents and 15 healthcare professionals. A thematic analysis was performed on the observations of A-V records and on the interview transcripts.

**Results:**

A total of 294 A-V records of the HELIX trial were analysed. Median (IQR) score for empathy, information and autonomy was 5 (0), 5 (1) and 5 (1), respectively. However, thematic analysis suggested that the consenting was a ceremonial process; and parental decision to participate was based on unreserved trust in the treating doctors, therapeutic misconception and access to an expensive treatment free of cost. Most parents did not understand the concept of a clinical trial nor the nature of the intervention. Professionals showed a strong bias towards cooling therapy and reported time constraints and explaining to multiple family members as key challenges.

**Conclusion:**

Despite rigorous research governance and consent process, parental decisions were heavily influenced by situational incapacity and a trust in doctors to make the right decision on their behalf. Further research is required to identify culturally and context-appropriate strategies for informed trial participation.

**Key questions**

**What is already known?**

- Informed parental consent for time-critical trials in neonatal intensive care units is challenging in high-income countries.
- Parental consent rates for neonatal intervention trials in low-and-middle-income countries are substantially higher than in high-income countries.

**What are the new findings?**

- Despite a rigorous informed consenting process, parental consenting was heavily influenced by an unreserved trust in the treating doctors and therapeutic misconception.
- Clinicians showed a clear bias favouring the intervention (cooling therapy), which was reflected in their communications with the parents.

**What do the new findings imply?**

- Ethical boards and research teams need to be cognisant of the possibility of systematic bias and therapeutic misconception in emulating successful models of treatments from high-income countries to low-and-middle-income countries.
- Culturally appropriate and innovative approaches to trial recruitment and consent seeking are needed for time-critical trials conducted in low-and-middle-income countries.

## Introduction

Informed parental consent for time-critical trials in neonatal intensive care units (NICUs) is challenging, as parents are likely to be under extreme emotional distress due to the critical clinical condition of their newborn infant.^1^ In such situations, the clinical or research staff who approach parents for trial participation require specific training, skills and empathy to assist parents in making an informed and voluntary decision about their baby’s participation in a trial. The depth and level of information provided in the consenting process is expected to be proportionate to the potential for harm and the vulnerability of the trial population; for example, interventional trials may require more discussion than observational studies.^2^

Parental consent processes for time-critical trials in low-and-middle-income countries (LMICs) involving populations from lower socioeconomic backgrounds are even more complex due to low literacy levels, parental disempowerment, hierarchies that exist in healthcare setting and larger societal factors.^3 4^ Furthermore, the research governance and ethical frameworks in LMICs are less rigorous and may not adequately protect the trial participants.^5^ Expensive interventions that are not easily available outside a LMIC trial setting may misguide participants by therapeutic misconception and false hopes.^6–8^ A recent systematic review comparing consent rates for 200 randomly selected recent neonatal interventional trial demonstrated significantly higher parental consent rates in LMICs (95.5%) when compared with high-income countries (HICs) (82.7%), raising concerns about the credibility of the consent processes in LMICs.^9^

We conducted a mixed-methods nested study within the ‘Hypothermia for encephalopathy in low and middle-income countries (HELIX) trial’ to assess the rigour of the informed consent process and understand the factors influencing parental decision-making to participate in the trial.

## Methods

The HELIX trial (ClinicalTrials.gov, NCT02387385), to which this mixed-methods study was linked, was an open-label, multi-country randomised controlled trial conducted in seven large public sector tertiary NICUs in India, Sri Lanka and Bangladesh. The trial was approved by the research ethics committees at Imperial College London and participating sites.

Prior to the trial launch, all the local clinical staff completed the International Conference on HarmonisationGood Clinical Practice certification and underwent rigorous training simulations on research ethics and informed consenting for the trial. There was specific emphasis during the training not to coerce parents into consenting for the trial by way of inducements such as financial benefits or specific investigations the baby may receive as a part of trial participation.

Between August 2015 and February 2019, parents of 475 term infants requiring an emergency admission to the NICU within 6 hours of birth due to neonatal encephalopathy were approached for trial participation.^10^ One of the neonatal physicians explained the trial procedures to the parents, shared a participant information leaflet (PIL) in the local language and then obtained written informed consent. Briefly, this included explaining the reason for conducting the trial, potential benefits and adverse effects of the intervention (cooling therapy), randomisation process, study investigations and the right to decline trial participation without affecting the baby’s clinical care in any way. Cooling therapy was administered using an approved servo-controlled cooling device (Tecotherm Neo) costing UIS$15 000, if the infant was randomised to the intervention group. All trial procedures were offered free of cost to all participants.

Audio-visual (A-V) recording of the consenting process was obtained in all cases to monitor that clinicians had provided all key information to parents, answered any questions they had and to ensure that parents were not coerced into consenting to the trial.

### Study design and participants

We used a qualitative dominant mixed-methods design. A quantitative assessment of the A-V recordings of HELIX trial’s consent process was undertaken by two investigators (SP and MAE) who were not involved in the original consenting process. A total of 294 A-V records of the HELIX trial which satisfied the quality parameters were analysed. Alongside, we used an exploratory observation method for capturing relevant aspects of consent process. Key observations and conversations between parents and professionals were noted down by SP and MAE independently.

**Figure 1:**
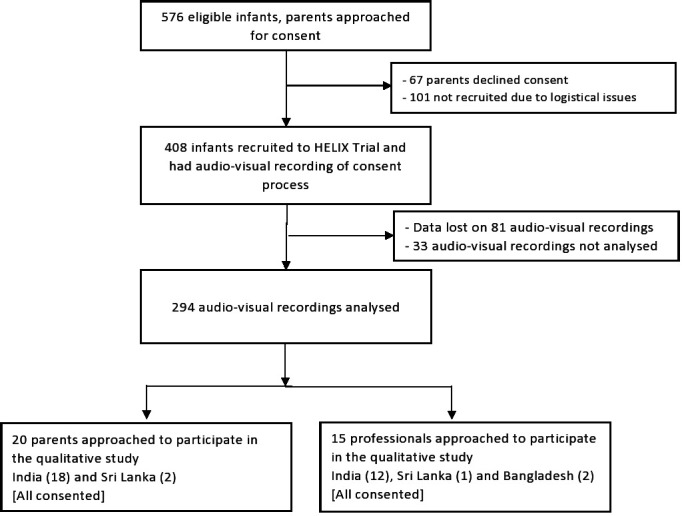
HELIX trial consent flow chart.

A subgroup of the 20 consecutive parents who had their child’s neurodevelopmental outcome assessments between August and September 2020 were invited to participate in a semi-structured in-depth interview, close to their clinical assessments at 18 months. Participants were recruited through purposive sampling; location (study sites) was considered to obtain diversity in narratives and experience. Parents from Bangladesh could not be interviewed due to language barrier and logistical issues. We interviewed medical and nursing teams (n=15) who were involved in the recruitment at all trial sites (figure 1).

Parental interviews explored their experiences prior to and after the birth of their baby, their decision of trial participation, understanding of the trial procedures and their overall experience of participation. Professional’s interviews were focused on the informed consent process, challenges faced during the process and their overall experience of recruiting to a time-critical neonatal randomised controlled trial. A total of 10 interviews were conducted face to face and 24 over telephone. The sample size was determined by data saturation, that is, at the point at which no new information or theme was observed in the data.^11^ The interviews were conducted by a female qualitative researcher (MAE). All interviews were audio recorded, translated and transcribed verbatim for analysis. NVivo V.12 software was used to organise and manage interview data.

### Data analysis

The A-V recordings of the original consent process were scored using a predefined proforma with 18 questions under 3 domains: (a) empathy^12^ (ie, respectful communication in local language and avoidance of medical jargon), (b) information (ie, explanation of all study procedures and risk benefits) and (c) autonomy^13^ (refer online supplemental file - annexure 1). Each question was scored using a 5-point Likert scale from 1 (lowest) to 5 (highest); median and IQR were calculated.

10.1136/bmjgh-2021-005757.supp1 Supplementary data

Interviews were analysed thematically, which involved reading the transcripts several times to identify, analyse and report the themes within and across the data.^14 15^ Analysis was broadly interpretive, and themes were inductively derived from the data. Constant comparative method was used to generate validity of data with an objective to inform ethical governance and guidelines in LMIC trials.^16^ SP and MAE conducted line-by-line coding of transcript and developed initial codes. Observation notes were also analysed thematically, which helped SP and MAE to reflect on interview findings and further refine the coding framework (refer online supplemental file - annexure 2). Finally, visual thematic maps were developed to conceptualise themes and find commonalities and the interdependence within these themes through an iterative process of concurrent reflection and discussion.

10.1136/bmjgh-2021-005757.supp2 Supplementary data

### Ethical considerations

Ethics approval for HELIX trial and the mixed-methods study was obtained from Imperial College Research Ethics Committee. The approvals included A-V recording of the consent process, review of A-V records as well as in-depth interviews with parents and professionals. The study objectives and voluntary nature of the study were explained to the participants and informed consent was obtained prior to the interviews.

### Patient and public involvement statement

Parent representatives from LMICs advised designing of HELIX trial as well as the PIL, although they were not directly involved in the design of the current study.

## Results

A total of 475 parents were approached, and 408 (86%) agreed to participate in the HELIX trial. Of these, 123 (30%) infants were born in the trial centres while 285 (70%) were either born in another facility or had home birth. The mean (SD) time from admission to the neonatal unit to consenting was 113 (97) min. Following parental consent, 202 infants were randomised to cooling and 206 to usual care.

### Analysis of A-V consent recordings

#### Semi-quantitative assessments

For HELIX trial, a total of 408 A-V records of parental consents were obtained in seven different South Asian regional languages. Neonatal consultants or the site principal investigator obtained the consent in 12% cases, and junior doctors in 88% cases, although this varied by the centre. Among these consent taking professionals, 53% were male and 47% were female. In most cases (89.3%), fathers were involved in consenting, followed by mothers (4.6%), both parents (3.1%) and relatives (3.1%). Mothers, when present, were passive observers, while fathers or other male family members present (in absence of the father) were seen engaging in discussions with the clinicians and making decision regarding child’s enrolment to the trial.

Due to issues such as loss of data and poor quality of audio, we were able to analyse a total of 294 A-V recordings from six centres. Mean (SD) duration of A-V recordings was 10.3 (7.2) min. Parents were briefed about key study information including randomisation, cooling therapy and risk–benefits in all cases. The information that was not discussed during the consent process included data confidentiality (12%), regulatory approvals (10%), additional blood tests and umbilical cord histology (9%), and the freedom to withdraw from the study at any time without the clinical care being affected (6%).

**Figure 2:**
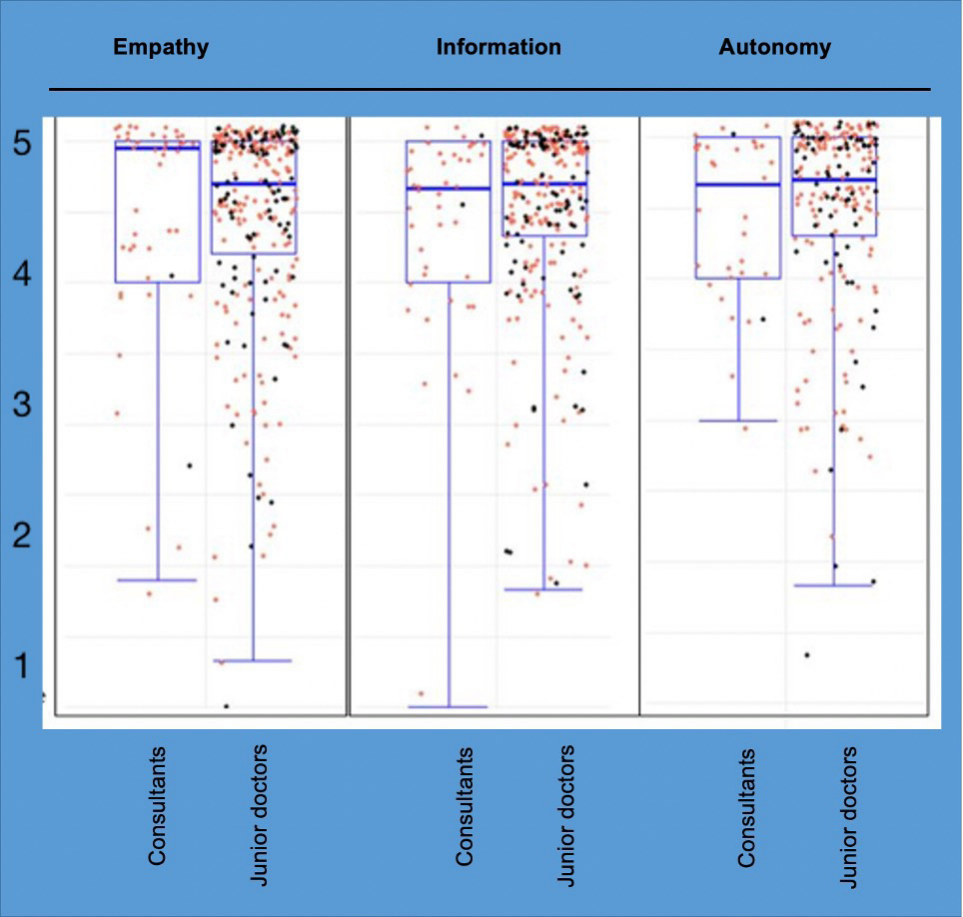
Distribution of the Likert scores under the three domains. Red dots indicate consent obtained during normal working hours (8:00 to 18:00) and black dots indicate consents obtained out of hours (18:00 to 20:00).

Semi-quantitative analysis based on the 17 predefined categories showed median (IQR) score for empathy, information and autonomy of 5 (0), 5 (1) and 5 (1), respectively, indicating an excellent informed consenting process. The consent quality scores of day-time and night-time recruits, and those obtained by the consultants and junior doctors were similar (p>0.05) (figure 2).

#### Thematic analysis of the A-V recording

The following themes were identified on careful observation of the A-V recordings of the consenting process.

### A ceremonial process

In most cases across study sites, the clinicians read out the PILs and parents rarely sought clarifications or asked questions. In most of the video records, clinicians were observed using phrases such as ‘*as I explained earlier*’ and ‘*as discussed with you*’, which indicated that the videos did not necessarily represent the first trial discussion with a family. The whole process appeared ceremonial; a task that needed to be fulfilled as per the trial protocol. Thus, the A-V recordings appeared to be a re-affirmation of the earlier discussion between the clinicians and the family.

### Clinician’s bias towards the intervention (cooling therapy)

Instances were observed where clinicians and research nurses were not in equipoise and referred to the trial treatment as something beneficial, which could help improve their baby’s outcome. As the following quotations illustrate, in some cases, clinicians highlighted access to free treatment through trial participation or made unsupported assurances about safety of cooling in this LMIC setting.

“It is your luck that you are here, outside for this treatment, you have to spend a lot of money. Here it is given free of cost”—Research nurse (Video analysis_355)

“Cooling will not cause any side effects, it is a proven treatment, and you need not worry about it”—Clinician (Video analysis_29)

### Disempowerment and trust in doctors to do ‘what was best’

Many parents seemed visibly distressed during recorded discussions and there were instances where fathers broke down during the consent process. In many recordings, parents told the doctor that they lacked sufficient knowledge to decide about trial participation and made requests for the doctor to do whatever they felt was best for their baby.

“We don’t know much, you know everything cos you are qualified, please see the baby and do whatever is necessary, they [referring centre] sent us here saying it will be good for the baby” (Video analysis_5)

“If you say so, it is fine, I will sign the paper. You know better as you are a doctor. We don’t even know A, B, C, D of this; we completely trust you and rest is up to God” (Video analysis_19)

### Analysis of the interviews with parents

A total of 20 parents were approached for the interviews and all of them consented to participate. Of these interviews, only fathers participated in 14, both parents in five and only mother in one. Parent profile is presented in table 1. Key themes developed from interviews with parents are given in the next section.

**Table 1:** Profile of parents interviewed

Participant characteristics	Classification	Number of parents (n=20)
Age (in years)	20–30	4
	31–40	14
	41–50	2
Education	Primary/secondary school	2
	High school	10
	Higher-secondary	4
	Undergraduate	4
Occupation*	Unemployed	1
	Skilled worker	10
	Semi-skilled worker	2
	Professional	1
	Self-employed	5
Monthly income (INR and USD)	<10 000 (<US$140)	4
	<20 000 (<US$285)	11
	<30 000 (<US$420)	2
	>30 000 (>US$420)	3

Semi-skilled workers included office helper, security personnel etc.

*Skilled workers included carpenters, drivers, mechanics, electricians, tailors etc.

**Limited understanding about the concept of a clinical trial and intervention: “Baby was kept on a machine”**

Despite AV recordings confirming that adequate information was provided to parents, interview findings indicated that most parents did not understand that they were participating in a clinical trial; parents felt that it as an additional treatment, for which they had to give consent. Many of them explained the trial as ‘*treatment was done using a machine*’ and that the treatment was provided by a ‘*team from London’*.

“The doctor told there is a chance for the baby to have some issues with the development, and his brain is weak, he needs to be put on the machine and his report will be sent to London and do you agree to do this. So I said yes and we agreed”—P4(3).

“When I came there they told that the baby is in a critical condition, there is a machine, they will keep the baby in the machine and computer will decide if the baby can be given this treatment or not”—P4(2).

**Making decisions under pressure: “We just wanted our baby to survive”:**

While reflecting on the decision to enrol their baby in the trial, most parents said that they were anxious and under tremendous pressure to decide about trial participation due to their baby’s critical condition. Many felt incapacitated by the situation, as they did not have any other option, but ultimately hoped that their child would survive.

“We were in great tension [stress], we were scared… we were thinking if this treatment will work or not, we were really worried, but what to do, we didn’t have any other option. We just wanted the best treatment to be done”—P2(3).

“That time we did not think much about the treatment we were only worried about the critical condition of our baby, and that she should get better. We did not think about anything else at that time”—P3(1)

Some accounts indicated that the unfamiliar hospital environment increased levels of fear and incapacity to make an informed decision. One father struggled to recall any details about the research, apart from how doctors reassured them that their child would survive if they took part in the trial.

“I don’t have any idea, I don’t know why they kept him. We were very scared we are from village and we don’t know anything about the hospital. I had gone for the first time and they kept saying that we have to do this for the baby and the baby will be alright and then we just told yes. We were really scared but still we told him to go ahead because our baby has to become alright”—P1(3)

**Cost concerns: “We did not have the money to go to private hospitals”:**

Treatment cost was another important factor which influenced parental decision-making. A few parents shared that they decided to participate in the trial once they learnt that the treatment and hospital care was offered free of cost.

“I said okay for everything… our friends told in [name] hospital and other private hospitals it [cooling therapy] will be about INR 4,00,000; in [name] hospital, it will be more than INR 10,00,000, but luckily the bed was available here”—P8(1)

“I didn’t have much money to go to the private, and when poor people like us go to the government hospital, we just trust God and then trust doctor. We are not rich, we are poor, so we told go ahead, we agree to what you are suggesting”—P3(3)

**Trust in treating doctors: “The doctors will do what is good”**

Parents had unreserved trust in doctors to do whatever was necessary to save their child’s life. Many appeared to believe the recruiting doctors were in a better position to decide about their own child’s trial participation. Parents described how they encouraged the doctor to do what they thought was appropriate by ‘letting the doctor get on with it’ without fully understanding what the trial entailed. Although PIL and most of the communication were in the local language, use of certain medical terminologies in English further limited their understanding of what the trial involved.

“I told the doctor to save my baby, whatever you feel appropriate you please do it. You are telling many things in English about medical things and I don’t understand those, but whatever you want to do, please go ahead with it”—P4(2)

“When the doctor says something, we will trust it 99%. We just assume that they are doing a good job and we let them do it”—P8(1)

**Parents’ belief in positive treatment outcomes: “Because we did this, baby is okay”**

Although the whole trial recruitment process was described to be quite stressful and confusing, most parents believed that their babies had a better outcome due to the machine treatment, and referred to their babies being ‘alright’, irrespective of adverse neurodevelopmental outcomes.

“I believed the doctor’s advice and now the baby is alright. At that time the advice given by the doctor satisfied me”—P3(2) (the child has cerebral palsy)

“Definitely the treatment has benefitted the baby. It was good, this treatment has to be given to more people”—P8(1) (the child has delayed speech development)

### Analysis of interviews with professionals

A total of 15 professionals were contacted and all of them consented to participate in the study. Of these, nine were neonatal trainees, two were consultants, and four were research nurses (RN) who assisted the recruitment and consent process (table 2). Key themes developed from interviews with professionals are shared in the next section.

**Table 2:** Profile of professionals interviewed

Participant characteristics	Classification	Number of respondents (n=15)
Gender	Male	5
	Female	10
Role in HELIX trial	Principal or co-investigator*	2
	Neonatal senior trainees (DM students)†	4
	Neonatal junior trainees (MD students)†	5
	Research nurses	4
Total years of experience	5–10 years	7
	11–15 years	6
	16–20 years	2

*Consultant staff.

†Junior doctors.

### Hesitation to use the term ‘trial’

Clinicians shared that they were comfortable in explaining or proposing the treatment and trial participation to parents as the treatment is a standard of care in most Western countries. Some expressed that they used the word ‘study’ and not ‘trial’ as they worried parents may consider this as an ‘*experimentation on babies*’ and may not agree to participate.

“We were really worried about what would be the response initially… we didn’t want to sound like they are some experimental guinea pigs; we were saying that it is a standard of care [in the UK], it is a study and it is well researched all over the world and we want to bring it to our country. I think the way you word it is very important, if you just tell them that it is part of a trial or a study then they may be worried”—Clinician 1(4)

### Limited parental understanding about randomisation

Clinicians described how most parents were from low socioeconomic backgrounds and had minimal levels of education. They believed it was often difficult for parents to comprehend their baby’s condition, as well as invitations to participate in the trial and details of the trial procedures. The concept of randomisation was described as being particularly difficult for parents to understand, with many parents insisting that their baby should receive cooling therapy, despite their babies being randomised to the control arm.

“Those who are a little more educated they probably understand what is happening better than who are living on the streets and less educated or doing some menial labour. Such parents could not understand, it was difficult for them to understand what was happening”—Clinician 1(3)

“Some parents would ask when the randomisation comes in the non-cooling arm, if there a possibility to do the cooling—such type of questions a few parents would ask. We will explain to them that whichever arm the randomisation comes to, we will be forced to do that and we will not be able to change it according to their wish”—Clinician 2(2)

Requests were perhaps unsurprising. The following quotes, as well as parent interview findings, suggest that parents’ requests to be allocated to the intervention arm appear to have been influenced by clinicians presenting the hypothermia treatment as proven to be beneficial for their baby.

“I think the main challenge was to explain to them about the concept of randomisation. That is the one thing they did not understand, ‘You told me that hypothermia is very beneficial and suppose by randomisation my baby doesn’t get it, then I am having a loss’, they would say”—Clinician 2(3)

“I wasn’t uncomfortable [taking consent] because it was not an experimental study or anything it is already a proven treatment. In western countries and many of the hospitals are doing it already. If you go to corporate hospitals, they will do it”—Clinician 1(1)

### Time pressure due to the narrow window period of recruitment

Within the limited therapeutic window of cooling therapy, clinicians often had less than an hour to counsel the parents about their baby’s condition, explain the trial and seek their consent. The pressure was multi-fold in cases where parents were not available to consent at the time of admission, as in case of outborn infants.

“Sometimes they would reach at 4 to 5 hours and then you have only one hour left within the time window. We have had one or two instances where we have started the process in just 10 minutes before the time period, time frame… So that was a challenge when they come from the far-off places”—Clinician1(4)

Many shared the view that such a short time was not enough for the parents to understand everything about the trial, especially considering their emotional distress.

“What I felt was most parents were in an emotional state to understand… asphyxia is an unexpected event. When they are referred from another hospital, it takes some time for this to sink in. We tell them that it is the study going on but sometimes it takes time for them to understand what really is happening”—Clinician1(4)

A few clinicians shared that owing to the parents’ requests, they often had to explain the trial information to multiple family members, and relatives, which further magnified time constraints.

“We have to explain individually like to 10 people, the same process again and again”—Clinician2(3)

### Misinformation given by referring centres

Clinicians reported how the complexities of explaining the trial to parents grew multi-fold when they were referred from peripheral centres with inaccurate explanations and expectations about the trial.

**“**They [the referring centre] wouldn’t have told them that there is 50% chance of either cooling or standard care. They would have just told that this is a new treatment and it is available in [hospital name]… please go, if you keep the baby here we are not sure what is going to happen. So many times they have come with that hope without any background knowledge”—Clinician 2(1)

“But one bad impact was there, some people just told that this is the new machine and you report over there, the baby will be cooled down”—Clinician2(5)

### Parental anxiety about A-V recording

While clinicians described the video recording as a good practice for standardising the informed consent process, it was a challenge logistically and often parents were apprehensive. Parents perceived that the treatment may not be safe and their consent to participation was recorded due to the risky nature of the trial. Due to this misguided belief, consent process was multi-layered and more time consuming, whereby clinicians would first inform parents about the trial, as well as video consent, clarify their queries and then be able to conduct the consent process again for the purpose of the video.

“Sometimes when we start video recording, they used to think—they are recording, and something is happening… they used to ask why you have to take a video like that. So, we used to explain to them that the video consenting is the ultimate form of consent”—Clinician 1(1)

“Something like this which is time critical and in a very emotionally charged situation… you’re trying to get the consent, in our set up, we can’t walk into the labour room with a video camera and then start the process. We have to formally say that we are going to do it with a written consent and then we do a video consent later on”—Clinician1(6)

## Discussion

In this study, we describe the unique complexities and challenges of informed consenting and the parental and professional perspectives of research participation in LMICs. Proforma-based quantitative analysis of A-V recordings suggested an elaborate consent process, where parents were provided adequate information prior to recruitment, including randomisation, potential risks–benefits and parental autonomy in decision to participate in the trial. On the contrary, observational analysis from A-V recordings clearly revealed clinician’s bias towards cooling therapy, and emotional distress and disempowerment of parents.

Parental interview findings were consistent with these observations. Decision to participate was primarily based on the trust in the treating doctors, and therapeutic misconception, and the opportunity to have an expensive treatment free of cost. Most parents did not understand the concept of a clinical trial nor the nature of the intervention. Lower levels of parental education and misinformation provided by the referring centres further convoluted the voluntary informed consent process. Interviews with professionals reiterated the strong bias towards cooling therapy, as this was already a standard of care in HICs and many LMIC hospitals in private sector. Time constraints and explaining to multiple family members were reported as the key challenges faced by professionals. In a situation of extreme distress, a positive validation of cooling therapy from professionals was a ‘ray of hope’ for parents. Therefore, parental decisions were not entirely autonomous and were influenced due to situational incapacity and a trust in doctors to make ‘the right decision on their behalf’.

Cooling therapy is recommended by the International Liaison Committee Resuscitation guidelines in 2015 as the standard of care in LMICs for neonatal encephalopathy.^17^ Although the guidelines acknowledged the poor-quality evidence regarding safety of cooling therapy in LMICs, it was already implemented in many NICUs in India^18^ and other South Asian countries^19^ as standard care, at the time of the HELIX trial recruitment. Hence, the bias of the clinicians towards cooling therapy during consenting is understandable. At the time of undertaking the qualitative study, HELIX trial was still ongoing, and hence clinicians were not aware of the trial outcomes. The trial has now been concluded and, contrary to the clinicians’ views, the results showed cooling therapy significantly increased mortality and offered no neuroprotection to infants in these settings.^20^ While these results were rather unexpected, it underlines the primary purpose of conducting any clinical trial—that is, to find out if a new treatment is beneficial or not. HELIX trial has reiterated the danger of directly extrapolating the results of research from high-income to low-income settings.

In HICs, research without prior consent has recently emerged as the preferred approach in time-critical trials, which allows the investigator to proceed with recruitment when treatment is required urgently and prior consent is not feasible.^21 22^ Some trials have sought parental assent prior to enrolment followed by a detailed informed consent to account for the emotional distress that parents experience affecting their ability to make informed decisions.^23^ However, in our view, research without prior parental consent is not an appropriate option in LMICs due to lack of rigorous research governance in these settings. Serious concerns have been reported time and time again about the adequacy of informed consenting processes in pharmaceutical-sponsored paediatric clinical trials in India.^24–26^ This issue prompted the Indian government to tighten regulatory approval of research and to mandate A-V recording of the informed consent process.^27^ While this was a step in the right direction, these regulations were subsequently removed by the Government of India due to the difficulties in obtaining such A-V recordings. Recent controversies about COVID-19 vaccination trials in India using financial incentives and inadequate informed consenting had led to international condemnation of the research governance.^28^ These malpractices are not uncommon in LMICs and therefore warrant a robust review and an oversight mechanism to protect vulnerable participants.

While our study provides useful insights into the consenting process followed in a neonatal time-critical trial in LMICs, it has certain limitations. First, although interview findings were supported by observations of A-V recordings and clinician accounts, parent interviews were conducted at follow-up visits approximately 18 to 22 months after original consenting and recruitment, which is likely to have impacted on recall. To minimise the recall bias, we interviewed parents who were recruited towards the end of trial. Second, information on parent’s sociodemographic information was not captured in HELIX trial. However, there is no reason to believe that the study sample of 20 parents is not a representative sample since recruitment was done consecutively. Third, in most cases the A-V recording seems to be done as a second or third layer of consenting, which precludes insight into initial recruitment discussions. The professionals reported that it was not easy for them to convince the parents to appear on the video recording and it required detailed explanation of the trial purpose and importance of recording the consenting process. They also reported that in certain cases where the baby was brought in at the very last minute, clinicians did not have the window to video record the consent. In such instances, A-V recording was done after the baby had been recruited to the trial. Hence, the analysis of consent videos may not have been a true depiction of consent process in all cases. Moreover, we interviewed only the parents of infants who survived. It is possible that those who had lost their infant had a different experience of trial participation. Finally, our study demonstrated an inadvertent contradiction between proforma assessments of A-V recordings and the observational as well as interview data highlight the inadequacy of quantitative assessments in such complex research questions and the need for in-depth qualitative enquiry to understand the rigour of consent processes in LMIC trials.

Better models for empowering parents and obtaining informed consent in LMIC needs to be carefully addressed in future trials. Innovative approaches using infographics and videos to communicate different aspects of trial may improve participant understanding and reduce anxiety,^29 30^ and such techniques may facilitate better informed consent in LMICs. Involving parents and family members in the design of studies would help in understanding their perspectives and addressing their priorities.

## Conclusion

Our findings highlight the challenges of obtaining a truly informed consent in LMICs, despite having a rigorous protocol for seeking consent as well as regular quality assurance audits. Extremely vulnerable and disempowered parents who lacked capacity to understand trial information and make an autonomous decision were influenced by the way the trial was presented to them. Lack of clinician equipoise and trial outcomes suggest that ethical boards and research teams need to be cognisant of the possibility of systematic bias and therapeutic misconception in emulating successful models of treatments from HICs to LMICs. Given the direct benefits of enhanced care anticipated by participants and their immense faith in the medical team, more efforts in finding culturally appropriate and innovative approaches to trial recruitment and consent seeking are needed for time critical trials conducted in LMICs.

## Data Availability

Data are available on request. Anonymised data can be provided on a case-by-case basis on request made to the corresponding author, for the purpose of future and further research.
