# Supplementary material for: Parental and professional perceptions of informed consent and participation in a time-critical neonatal trial: a mixed-methods study in India, Sri Lanka and Bangladesh
Source: BMJ Glob Health. 2021 May 21;6(5):e005757. doi: 10.1136/bmjgh-2021-005757 (PMC8144040; doi:10.1136/bmjgh-2021-005757)
Supplement: Supplementary data [file bmjgh-2021-005757supp001.pdf]

HELIX Number	
Date of consent	
Time of consent	
Person taking consent (Consultant/PG student)	
Trainee/PI	
Research nurse present (Y/N)	
Consent outcome (Given/Refused)	
Outcome of baby (survived/died)	
VIDEO SCORING	
EMPATHY	
1. Dr showed empathy and respect to the parents	
2. Dr used a regional language understood by parents and avoided medical jargon	
INFORMATION	
3. Dr explain why the baby has been selected for the study	
4. Dr explained the purpose of the study	
5. Dr explained all the study procedures - details of Cooling therapy and duration	
6. Dr explained all the study procedures - infection test from blood and umbilical cord	
7. Dr explained all the study procedures - MRI scan within 2 weeks	
8. Dr explained all the study procedures - Follow up at 18 months	
9. Dr explained randomisation (50:50 chance of cooling or usual care decided by a computer)	
10. Dr explained the potential benefits of the study	
11. Dr explained the potential risks of the study	
12. Dr explained how the research costs will be covered and explain any renumeration	
13. Dr explained that partipation was voluntary, non-participation would not affect the care; and avoid use of any coercion	
14. Dr explained parents could withdraw from the study any time without giving any reason	
15. Dr explained how confidentiality of the data (names etc) will be maintained	
16. Dr provided information about the ethics and regulatory approvals of the study	
AUTONOMY	
17. Dr encouraged the parents to ask questions and then respond adequately	
18. Parents asked appropriate questions (if so, please please record the questions asked by parents)	
