# Supplementary material for: Parental and professional perceptions of informed consent and participation in a time-critical neonatal trial: a mixed-methods study in India, Sri Lanka and Bangladesh
Source: BMJ Glob Health. 2021 May 21;6(5):e005757. doi: 10.1136/bmjgh-2021-005757 (PMC8144040; doi:10.1136/bmjgh-2021-005757)
Supplement: Supplementary data [file bmjgh-2021-005757supp002.pdf]

Supplementary file- annexure 2

(A) Coding framework for parent interviews

Root nodes	Parent nodes	Child nodes
Trial participation	Delivery complications	Prolonged labour
		Forceps delivery
		Delayed cry of baby
	Decision making	Emotional distress
		Cost concerns
		Trust in doctors
		Time constraints
		Level of education
	Parents understanding of trial	Additional treatment
		Treatment using a machine
		Treatment from abroad
	Follow up	Doctors explained what to expect
		Unsure about Bayley test details and result
Views on trial	Positive outcome	Baby became better because of the treatment (cooling)
		Baby is alright (irrespective of developmental delays)
		Doctors communication
	Video consenting	Anxiety of parents
		Perception of treatment to be risky
		AV records sent to London

(B) Coding framework for professional's interview

Root nodes	Parent nodes	Child nodes
Preparation for trial	Training	GCP certification
		Role plays
		Observation of consenting by seniors
		Documentation
		Technical – operating Tecotherm machine
Consent process	Parents profile	Lower education levels
		Poor socio-economic backgrounds
		Migrant labourers
	Declining consent	Distance from trial sites
	Queries from parents	Survival of the baby
		Long term effects
		Cooling vs keeping baby warm
		Side effects
	Presenting the trial	Trial as a study
		Presented as management system
Challenges in consenting	Time constraints	Delay in referral

		Parents not available
		Night admissions
	Introducing the trial to parents	Parents not understanding trial terms- randomisation, follow up
	Explaining to multiple family members	Parents bringing relatives, grand parents
	Information given by referral hospital	Additional treatment facility
		Cooling as a definite therapy
	Randomisation	Explaining the concept of randomisation
		Parent demanding cooling
	Video consenting	Parents anxiety
		Technical glitches
		Parents not available
		Repeat the same information in front of camera

(C) Coding scheme for observation of AV records

Root nodes	Parent nodes	Child nodes
Consent process	Explanation of baby's condition	Long term and short terms effects including mortality rates
	Introducing the trial	Treatment from London
		Standard of care in West
		Free treatment
		Follow up
		Travel assistance for follow up
Doctors communication	Use of medical terms and jargons	Encephalopathy
		Hypothermia
		Randomisation
	Reading from PIL	Sharing the PIL with parents
		Allowing time to read in case parents wish
	Cooling to be beneficial	No side effects to mild side effects
		Baby will benefit from cooling
		Cost factors
Parents participation	Emotional state	Distressed (crying)
		Shocked
		Numb
	Queries and doubts	Survival of the baby
		Long term effects of cooling
		Follow up
	Mothers (female members)	Passively listening
		Seeking time to discuss with male members
